# Black Goji Berry (*Lycium ruthenicum*) Juice Fermented with *Lactobacillus rhamnosus* GG Enhances Inhibitory Activity against Dipeptidyl Peptidase-IV and Key Steps of Lipid Digestion and Absorption

**DOI:** 10.3390/antiox13060740

**Published:** 2024-06-19

**Authors:** Kritmongkhon Kamonsuwan, Vernabelle Balmori, Marisa Marnpae, Charoonsri Chusak, Thavaree Thilavech, Suvimol Charoensiddhi, Scott Smid, Sirichai Adisakwattana

**Affiliations:** 1 Center of Excellence in Phytochemical and Functional Food for Clinical Nutrition, Department of Nutrition and Dietetics, Faculty of Allied Health Sciences, Chulalongkorn University, Bangkok 10330, Thailand; 6278303837@student.chula.ac.th (K.K.); charoonsri.c@chula.ac.th (C.C.); 2 Department of Food Science and Technology, Southern Leyte State University, Sogod 6606, Philippines; vbalmori@southernleytestateu.edu.ph; 3 The Halal Science Center, Chulalongkorn University, Bangkok 10330, Thailand; marisa.m@chula.ac.th; 4 Department of Food Chemistry, Faculty of Pharmacy, Mahidol University, Bangkok 10400, Thailand; thavaree.thi@mahidol.edu; 5 Department of Food Science and Technology, Faculty of Agro-Industry, Kasetsart University, Bangkok 10900, Thailand; suvimol.ch@ku.th; 6 Discipline of Pharmacology, School of Biomedicine, Faculty of Health and Medical Sciences, The University of Adelaide, Adelaide 5000, SA, Australia; scott.smid@adelaide.edu.au

**Keywords:** black goji berry, *Lactobacillus rhamnosus*, anthocyanins, flavonoids, non-volatile metabolite profiling

## Abstract

With the global increase in hyperglycemia and hyperlipidemia, there is an urgent need to explore dietary interventions targeting the inhibition of dipeptidyl peptidase-IV (DPP-IV) and lipid digestion and absorption. This study investigated how *Lactobacillus rhamnosus* GG (LGG) affects various aspects of black goji berry (BGB) (*Lycium ruthenicum* Murr.) juice, including changes in physicochemical and functional properties, as well as microbiological and sensory attributes. Throughout the fermentation process with 2.5–10% (*w*/*v*) BGB, significantly improved probiotic viability, lactic acid production, and decreased sugar content. While total flavonoids increase, anthocyanins decrease, with no discernible change in antioxidant activities. Metabolite profiling reveals elevated phenolic compounds post-fermentation. Regarding the inhibition of lipid digestion and absorption, fermented BGB exhibits improved bile acid binding, and disrupted cholesterol micellization by approximately threefold compared to non-fermented BGB, while also increasing pancreatic lipase inhibitory activity. Furthermore, a decrease in cholesterol uptake was observed in Caco-2 cells treated with fermented BGB (0.5 mg/mL), with a maximum reduction of 16.94%. Fermented BGB also shows more potent DPP-IV inhibition. Sensory attributes are significantly improved in fermented BGB samples. These findings highlight the potential of BGB as a bioactive resource and a promising non-dairy carrier for LGG, enhancing its anti-hyperglycemic and anti-hyperlipidemic properties.

## 1. Introduction

One of the most concerning health challenges globally is the increasing incidence of non-communicable diseases (NCDs). These persistent conditions, which are not contagious, present a substantial global health concern requiring urgent attention and collaborative effort. Hyperlipidemia and hyperglycemia are crucial contributors to the development and progression of NCDs such as cardiovascular diseases, stroke, and diabetes [1]. The high prevalence of hyperlipidemia is a concerning global health issue, contributing significantly to morbidity and mortality rates worldwide [2]. There is a current trend towards investigating novel methods to address hyperlipidemia beyond traditional approaches. One promising approach to managing hyperlipidemia involves targeting key steps in lipid digestion and absorption. Recent research suggests that inhibiting pancreatic lipase, cholesterol micellization, and bile acid reabsorption could be effective. These strategies aim to reduce dietary fat absorption, potentially lowering circulating lipid levels and decreasing the risk of cardiovascular disease [3]. Elevated blood glucose levels, characteristic of diabetes, are associated with severe complications including cardiovascular disease, neuropathy, nephropathy, and retinopathy. Effective management of hyperglycemia is imperative in diabetes care to mitigate these adverse outcomes. One treatment strategy involves targeting the enzyme dipeptidyl peptidase-IV (DPP-IV), which is pivotal in regulating incretin hormones like glucagon-like peptide-1 (GLP-1) and glucose-dependent insulinotropic polypeptide (GIP). Inhibiting DPP-IV decreases the breakdown of these incretin hormones, extending their effects. This leads to increased insulin secretion, decreased glucose production, and consequently, better blood sugar management. Current research has demonstrated that DPP-IV inhibitors, such as sitagliptin and vildagliptin, are effective in improving glycemic control in diabetic patients [4]. Aligned with these strategies, significant focus has been placed on utilizing probiotics and functional foods, including phytochemicals, to regulate lipid and carbohydrate metabolisms and support cardiovascular health. Moreover, probiotics and phytochemicals have been found to exhibit both lipid-lowering and glucose-lowering effects, making them promising adjuncts in the management of metabolic disorders [5,6].

Probiotic-fermented fruit juice has become a popular and commercially available food product, drawing attention from a broad spectrum of consumers, including those with dietary restrictions [7]. The probiotic fermentation process not only extends shelf life but also enhances biological properties and improves sensory attributes [8]. Probiotic enzymes are integral in breaking down diverse compounds within fruit matrices, resulting in the generation of active substances such as polysaccharides, organic acids, short-chain fatty acids, and phenolic compounds. Concurrently, the fermentation process decreases sugar content and reduces antinutritional factors like alkaloids, tannins, and oxalates [9]. These bioconversions could transform the functional components of fruit juice together with enhancing their bioaccessibility and bioavailability. Furthermore, the probiotic-mediated bioconversion contributes to increased antimicrobial, anticancer, and antioxidant activities in food products, generating esters, alcohols, and terpenes that improve sensory quality [10,11].

The genus *Lactobacillus*, a subset of lactic acid bacteria (LAB), stands out as a widely utilized category of probiotics, recognized for its “generally recognized as safe” (GRAS) status and potential health advantages [12]. Specifically, *Lactobacillus rhamnosus* GG (LGG), the initial patented strain within the genus, emerges as a potential probiotic with the capacity to endure and thrive in both acidic and alkaline environments, exhibiting robust growth characteristics, and an ability to adhere to the intestinal epithelial layer [13]. These attributes position LGG favorably for application in the industry-level production of fermented foods. Extensive research has demonstrated the health-promoting effects of LGG, in both in vitro and human studies [14]. Notably, LGG has shown efficacy in safeguarding the mucosa, bolstering intestinal crypt survival, and fostering immune responses. Moreover, it has been reported to alleviate conditions such as diarrhea, gastrointestinal infections, irritable bowel syndrome, and inflammatory bowel disease [15]. Consequently, LGG emerges as a promising strain for the development of food products with potential health benefits.

Various fruits, including pomegranate, cherry, mulberry, blackberry, blueberry, and goji berry, serve as carriers for probiotics [16]. These non-dairy carriers are renowned for their abundance of phytochemical compounds, which possess potent antioxidant activities. Within the goji berry family (*Lycium* spp.), black goji berry (*Lycium ruthenicum* Murr.) (BGB) emerges as a particularly promising raw material for fermented fruit juice production. Specifically, it has been identified to exhibit higher phytochemical and antioxidant activities compared to other goji berry varieties, such as red goji berry [17]. When directly compared to other berry fruits like blackberry or blueberry, BGB demonstrates elevated anthocyanin contents, along with other phytochemical compounds such as flavonoids, phenolic acids, and carotenoids [18]. These phytochemical components contribute to various biological properties and health benefits in BGB, similar to those found in fermented food products, including anti-diabetic, anti-hyperlipidemic, and prebiotic-like properties [19].

Despite these promising attributes, there is limited evidence supporting BGB juice as a carrier for probiotics, especially *Lactobacillus* spp. Thus, this study aimed to investigate the impact of LGG on the fermentation process of BGB juice. Furthermore, it explored changes in physicochemical properties, non-volatile compounds, antioxidant capacity, probiotic viability, and sensory attributes. Additionally, the study assessed the effects on key steps of lipid digestion and absorption, including pancreatic lipase inhibition, cholesterol micelle formation, cholesterol uptake in enterocytes, and bile acid binding. Moreover, it examined the inhibitory effects of BGB and its fermentation on DPP-IV activity. The findings hold promise for the development of novel fermented juice with potential health benefits, particularly in modulating DPP-IV activity and lipid digestion and absorption.

## 2. Materials and Methods

### 2.1. Materials

The BGB was commercially purchased from a reputable Chinese dispensary (Chin Heng Huat, Bangkok, Thailand). The starter culture was a commercial probiotic *Lactobacillus rhamnosus* GG, obtained from Chr. Hansen A/S, Horsholm, Denmark, in freeze-dried direct vat set (DVS) format. Folin–Ciocalteu reagent, 1,1-diphenyl 2-picrylhydrazyl (DPPH), ascorbic acid, 6-hydroxyl- 2,5,7,8-tetramethylchromane-2-carboxylic acid (Trolox), 2-2′-Azino-bis (3-ethylbenzothiazoline-6-sulfonic acid) (ABTS), 2,4,6-tripyridyl-s-triazine (TPTZ), phosphatidylcholine, and oleic acid were purchased from Sigma-Aldrich Chemical (St. Louis, MO, USA). Cholesterol test kits (Cholesterol liquicolor^®^) were obtained from Human Diagnostics (Wiesbaden, Germany), and the total bile acid kit was purchased from BIOBASE (Jinan, China). The DPP-IV Inhibitor screening assay kit was procured from Cayman Chemical (Ann Arbor, MI, USA), while the *Lactobacillus* MRS agar was obtained from Himedia (Thane, India). Caco-2 cells were obtained from the Sigma-Aldrich (Sydney, NSW, Australia). Ezetimibe was purchased from Merck, Sharp, & Dohme (Kenilworth, NJ, USA). 22-(N–N7-nitrobenz-2-oxa-1,3-diazol-4-yl) amino)-23,24-bisnor-5-cholen-3-ol (NBD-cholesterol) was obtained from Invitrogen (Eugene, OR, USA).

### 2.2. Preparation of BGB Powder

The extraction method was modified from the procedure outlined in a previous study [17]. Dried fruit was boiled with distilled water at a concentration of 5% (*w/v*) within a temperature range of 95 to 100 °C for 60 min. The resulting aqueous solution of BGB was filtered through Whatman 125 mm filter paper. Subsequently, the sample underwent lyophilization using a freeze-drying technique (Hong Ta Enterprise, Samutprakarn, Thailand). The BGB powder was then stored at −20 °C for further investigation.

### 2.3. Fermentation

For the fermentation process, prepared BGB powder was employed to create infusions with concentrations of 2.5%, 5%, and 10% *w*/*v*, supplemented with 5% *w*/*v* refined sugar. Following pasteurization conditions outlined in a prior study at 65 °C for 30 min [20], LGG freeze-dried granules were directly added to the pasteurized BGB infusion at a concentration of 0.005% *w*/*v* inoculation to reach the bacterial concentration at 7 log CFU/mL. Then, slow agitation for 30 min ensured even distribution of the culture. BGB samples were transferred in the air-tight glass jar under anaerobic conditions. The incubation temperature was maintained at 37 °C for 24 h in a digital incubator (DAIHAN Scientific, Wonju, Republic of Korea). Samples were taken at 0 and 24 h fermentation for further analysis. The process of lyophilization, using a freeze-drying method, was conducted on fermented samples in preparation for the subsequent cholesterol uptake assay in the Caco-2 cell line.

### 2.4. Determination of Physicochemical Properties

#### 2.4.1. pH, Total Soluble Solid (TSS), and Color

pH measurements were performed using a pH meter (S40 Seven MultiTM, Metter-Toledo, Switzerland). Total Soluble Solids (TSS) were determined with a hand-held refractometer (HSR-500, Atago Co. Ltd., Tokyo, Japan), and the results were expressed as °Brix. Color measurements were conducted using a colorimeter (CM-3500D, Konica Minolta Co. Ltd., Tokyo, Japan), with results expressed as L* (Lightness/Brightness), a* (Greenness to Redness), and b* (Blueness to Yellowness). To ensure accuracy, all samples underwent triplicate measurements, and the calibration process adhered to the protocols specified for each method.

#### 2.4.2. Total Carbohydrate and Reducing Sugar

Total carbohydrates were determined using the phenol-sulfuric acid method while reducing sugars were analyzed using the DNS method [21]. For total carbohydrate analysis, 400 μL of the sample was mixed with 10 μL of 80% phenol (%w/w) and 1 mL of sulfuric acid. After a 10 min stand and cooling at room temperature, absorbance was read at 490 nm. Total carbohydrate content was calculated using a glucose standard curve and expressed as mg/mL sample.

To analyze reducing sugars, 250 μL of the sample was mixed with 250 μL of DNS reagent (1% 3,5-dinitrosalicylic acid, 0.2% phenol, 0.05% Na_2_SO_3_, and 1% NaOH in an aqueous solution). The mixtures were heated at 100 °C for 10 min to stop the reaction. Then, 250 μL of 40% potassium sodium tartrate solution was added to stabilize the color, and absorbance was recorded at 540 nm. Glucose solution served as a standard, and the reducing sugar content was expressed as mg glucose equivalents/mL sample.

#### 2.4.3. Analysis of Lactic Acid

The lactic acid concentration was determined using a previously published method [22]. The BGB sample was centrifugated at 6500× *g* for 20 min at 4 °C and filtered through a 0.2 μm PVDF syringe filter. Subsequently, 5 μL of the sample was injected into a Nexera UHPLC System (Shimadzu, Kyoto, Japan) equipped with an Inertsil^®^ ODS-3 C_18_ reverse-phase column (250 mm × 4.6 mm, 5 μm) at 35 °C. The mobile phase, consisting of 10 mM KH_2_PO_4_ (pH 2.4) with phosphoric acid (mobile phase A) and acetonitrile (mobile phase B), flowed at a rate of 0.7 mL/min. The gradient program was set as follows: 0.01–7 min, 25% B; 8–11 min, 50% B; 12 min, 25% B, held until 15 min, with UV–visible detection at 210 nm. Identification of results was achieved by comparing peak retention times with standards, and quantification employed the external standard method. 

#### 2.4.4. Total Phenolic Content (TPC)

Briefly, 50 μL of the sample was mixed with Folin–Ciocalteu reagent, which had been diluted 10× in distilled water, and allowed to stand for 10 min. Then, 50 μL of Na_2_CO_3_ was added, and the absorbance was measured at 760 nm after a 30 min incubation in the dark at room temperature. The results were determined using a standard curve of gallic acid and expressed as g gallic acid equivalent (GAE)/100 mL [23].

#### 2.4.5. Total Anthocyanin Content (TAC)

TAC was determined using the pH differential method [24]. Two buffer systems, 0.025 M potassium chloride at pH 1.0 and 0.4 M sodium acetate at pH 4.5, were prepared. Subsequently, 500 μL of the sample was mixed with each buffer (500 μL), and the absorbance at 520 nm and 700 nm was measured against a distilled water-filled blank cell. The results were expressed as mg cyanidin-3-glucoside equivalent (C3GE)/100 mL. Anthocyanin content (*c*) was calculated following the formula (Equations (1) and (2)):

(1) $$∆A=\left(A_{520nm}-A_{700nm}\right)pH1.0-\left(A_{520nm}-A_{700nm}\right)pH4.5$$

(2) $$c\left(mg/100\;mL\right)=(∆A\times MW\times DF\times 10^{3})/\varepsilon \times 1\;\;$$

where *MW* represents the molecular weight of cyanidin-3-glucoside (449.2 g/mol), *DF* represents the dilution factor, 10^3^ represents the factor for conversion from g to mg, *ɛ* represents extinction coefficients of cyanidin-3-glucoside (26,900 L/mol·cm), and l represents pathlength in cm.

#### 2.4.6. Total Flavonoid Content (TFC)

TFC was assessed following a published method [25]. A 50 μL sample aliquot was mixed with 10 μL 10% *w*/*v* AlCl_3_ solution, 10 μL 1 M sodium acetate, and 150 μL absolute ethanol. After a 30 min incubation, the absorbance was measured at 430 nm, and the results were expressed as mg quercetin equivalent (QE)/100 mL.

#### 2.4.7. Antioxidant Activities

##### Ferric-Reducing Antioxidant Power (FRAP)

The antioxidant potential of the sample was measured using the FRAP assay [26]. The FRAP reagent, composed of 0.3 M sodium acetate buffer solution (pH 3.6), 10 mM TPTZ solution in 40 mM HCl, and 20 mM FeCl_3_ solution in a 10:1:1 ratio, was mixed with 10 μL of samples. After a 30 min incubation in the darkness at room temperature, the absorbance was recorded at 595 nm. The results were calculated using a FeSO_4_ standard curve and expressed as mmol FeSO_4_/100 mL.

##### DPPH Radical Scavenging Activity

The DPPH radical scavenging activity was determined using the stable radical DPPH (2,2-diphenyl-1-picrylhydrazyl) as described previously [26]. In summary, 10 μL of the sample was mixed with 90 μL of 0.2 mM DPPH reagent (diluted with ethanol). Ascorbic acid (1 mg/mL) served as the standard. After a 30 min incubation at room temperature, the absorbance was measured at 515 nm. The results were expressed as mg ascorbic acid equivalent (AAE)/100 mL.

##### Trolox Equivalent Antioxidant Activity (TEAC)

For TEAC, the method adapted from a previous publication that induced the radical anion (ABTS^°+^) by adding 2.4 mM potassium persulfate (K_2_S_2_O_8_) and 7 mM ABTS [26]. After 16 h of dark incubation, 10 μL of the sample was mixed with 90 μL of ABTS^°+^ solution. The decrease in solution absorbance was measured at 734 nm. The results were calculated using the Trolox standard curve and expressed as mg Trolox equivalent (TE)/100 mL.

### 2.5. Non-Volatile Metabolite Profiling Using Liquid Chromatography Coupled with High-Resolution Fourier Transform Mass Spectrometry (LC-HRFTMS)

Untargeted metabolomics is a highly utilized approach for studying variations in the metabolite composition of plants. This method involves analyzing all metabolites present in a biological sample without specific targeting, allowing for a comprehensive understanding of the diverse metabolic activities within plants [27]. Using the tools of metabolomics through the employment of LC-HRFTMS is an efficient approach. Goji berry samples underwent Solid Phase Extraction (SPE) to enhance metabolite detection, followed by separation on a Thermo Vanquish Horizon LC system coupled to a Thermo Orbitrap IDX HR-FTMS (Thermo Fisher Scientific, Waltham, MA, USA). MS and MS/MS analyses were conducted in both negative and positive electrospray ionization (ESI) modes. The Kinetex F5 column (2.6 µm, 150 mm × 2.1 mm ID) provided steric selectivity for separating structural isomers. Key settings included a 2 μL injection for MS analysis and 4 μL for MS/MS analysis, a flow rate of 0.4 mL/min, and an oven temperature of 30 °C, with a total LC runtime of 42 min. The mobile phase, composed of 0.1% formic acid, 0.5% Methanol in Milli-Q water (A), and 0.1% formic acid, 2% Milli-Q water, and 40% Acetonitrile in Methanol (B), followed a specific gradient: 0.00–6.25 min, 0–1% B; 6.25–20.00 min, 1.0–7.5% B; 20.00–30.00 min, 7.5–60% B; 30.00–33.00 min, 60–90% B; 33.00–38.00 min, 90% B; 38.00–38.20 min, 90–0% B, and held until 42 min. The ESI interface featured a +3500 V capillary, an ion transfer tube temperature of 275 °C, and a vaporizer temperature of 300 °C. The auxiliary gas flow was set to 7 units with a sheath gas flow of 25 units. This method, paired with the specified MS conditions, enables the capture of a diverse range of slightly polar to non-polar non-volatile molecules. Putative identification of the compounds was assigned based on internal and external spectral databases.

### 2.6. Determination of Biological Activities

#### 2.6.1. Bile Acid Binding Activity

The bile acid binding ability assay was conducted with minor adjustments, following a previous publication [25]. Bile extract (1 mg/mL) in 0.1 M phosphate-buffered saline (PBS), pH 7.4) served as the bile acid in this experiment. Cholestyramine (3 mg/mL) was employed as a positive control. Incubation of the samples with bile extract occurred at 37 °C for 90 min. The resulting mixture underwent filtration through a 0.22 μm syringe filter. The collected filtrate was then analyzed spectrophotometrically at 540 nm using a bile acid analysis kit.

#### 2.6.2. Pancreatic Lipase Inhibitory Activity

The inhibitory effect on pancreatic lipase was investigated following a previously established protocol [25]. The 5 μL of samples were mixed with 45 μL of pancreatic lipase solution (7.5 mg/mL). Subsequently, 50 μL of 4-methylumbelliferone oleate (4-MUO) solution (0.2 mM in 0.1 M PBS, pH 7.0) was added to initiate the enzyme reaction. The incubation was carried out at 37 °C for 20 min. To halt the enzymatic reaction, 100 μL of 0.1 M sodium citrate (pH 4.2) was introduced. Orlistat served as the positive control. The amount of 4-methylumbelliferone by the lipase was quantified using fluorescence spectroscopy with an excitation wavelength of 320 nm and an emission wavelength of 450 nm. 

#### 2.6.3. Cholesterol Micellization Inhibitory Activity

To establish a cholesterol solubilization model, artificial micelles were generated based on a previously conducted study [25]. In brief, a mixture of 2 mM cholesterol, 1 mM oleic acid, and 2.4 mM phosphatidylcholine in methanol was prepared. The mixture was dried under nitrogen gas. Then, 15 mM PBS (pH 7.4) with 6.6 mM taurocholate salt was redissolved. The mixture underwent sonication before incubating at 37 °C overnight. Following this, BGB samples were introduced to the artificial micelle solution and incubated for 120 min at 37 °C. Cholesterol content was assessed using cholesterol test kits, with gallic acid employed as the positive control. The results were expressed as the percentage inhibition of cholesterol micellization. 

#### 2.6.4. Dipeptidyl Peptidase-IV (DPP-IV) Inhibitory Activity

DPP-IV inhibitory activity was assessed utilizing a DPP-IV inhibitor screening assay kit, employing a fluorescence-based method for screening inhibitors. In brief, 10 μL of the samples were added to a 96-well plate containing 30 μL of diluted assay buffer and 10 μL of diluted DPP(IV). The reaction was initiated by adding 50 μL of diluted substrate solution, followed by incubation at 37 °C for 30 min. The fluorescence intensity, with an excitation of 355 nm and an emission wavelength of 460 nm, was monitored using a spectrofluorometer. Sitagliptin was used as the positive control.

### 2.7. Microbiological Analysis

The microbiological analysis for this study was based on the U.S. Food and Drug Administration’s (U.S. FDA) Laboratory Method [28]. The number of microorganisms in the products was evaluated using the aerobic plate count (APC) technique. Viable cells of lactic acid bacteria were quantified using MRS (De Man, Rogosa, and Sharpe) agar. Samples were diluted with peptone solution (0.1% *w*/*v*). All equipment and materials were sterilized using an autoclave at 121 °C for 15 min. The dilutions were shaken and subjected to the pour plate technique using warm MRS agar. Plates were incubated at 37 °C for 48 h and colonies were counted using a colony counter.

### 2.8. Caco-2 Cell Culture

#### 2.8.1. Cell Viability

The cytotoxic effects of BGB were evaluated using the 3-(4,5-dimethylthiazol-2-yl)-2,5-diphenyltetrazolium bromide (MTT) colorimetric assay. Caco-2 cells, a human epithelial colorectal adenocarcinoma cell line, were employed for this study. Cells were seeded in 96-well plates at a density of 2 × 10^4^ cells/mL and incubated overnight in Dulbecco’s Modified Eagle’s Medium (DMEM) supplemented with 10% fetal bovine serum (FBS), 1% non-essential amino acid (NEAA), and 1% penicillin–streptomycin solution (Complete medium). Following adherence, the cells were exposed to varying concentrations of BGB (ranging from 0.025 to 2 mg/mL) or vehicle control for 24 h. Subsequently, the culture medium was removed, and the cells were incubated with 0.25 mg/mL MTT solution (prepared in serum-free DMEM) for 3 h at 37 °C in a humidified atmosphere containing 5% CO_2_. The formazan crystals formed by the reduction of MTT by metabolically active cells were solubilized by adding 100 μL of dimethyl sulfoxide (DMSO) to each well. The absorbance was measured at 570 nm using a microplate reader. The results were presented as the percentage of cell viability compared to the untreated cells (% of control).

#### 2.8.2. Cholesterol Uptake in Caco-2 Cells

Caco-2 cells were maintained in complete DMEM high glucose. Cells were passaged every 3–4 days upon reaching approximately 80% confluence. Cells were seeded onto 24-well plates at a density of 25,000 cells per well and then incubated at 37 °C for 7 days to allow for differentiation. During this period, the medium in the well was replaced every 2 days. Experiments were carried out following the previous publication with slight modifications [29]. After the differentiation period, cells were starved with serum-free DMEM low glucose for 24 h. Subsequently, cells underwent two washes with Hanks’ balanced salt solution (HBSS; composed of 140 mM NaCl, 5 mM KCl, 1.2 mM Na_2_HPO_4_, 2 mM CaCl_2_, 1.2 mM MgSO_4_, 20 mM HEPES, and 0.2% bovine serum albumin, pH 7.4) and were then incubated in HBSS at 37 °C 1 h before the experiments. After incubation, cells were treated with free cholesterol (containing 0.5 mM taurocholate salt and 25 μM NBD-cholesterol) supplemented with varying concentrations of BGB (0.05–0.5 mg/mL). Ezetimibe (50 μM) served as a positive control. Cells underwent five washes with cold HBSS, and the fluorescence was measured at an excitation wavelength of 485 nm and an emission wavelength of 535 nm. Cell lysis was performed using a buffer solution (composed of 10 mM tris-HCl pH 7.4, 150 mM NaCl, 1% Triton-X-100, 1 mM EDTA, and 0.1% SDS), employing two freeze–thaw cycles. Lysates were then centrifuged at 12,000 rpm at 4 °C for 10 min, and supernatants were collected for protein concentration determination using a BCA kit (Thermo Fisher Scientific, Waltham, MA, USA), with bovine serum albumin as the standard. Total protein quantification enabled normalization based on the total number of cells used. Results were expressed as the percentage of cholesterol uptake relative to control values.

### 2.9. Sensory Evaluation

The sensory quality of the samples was assessed by a panel of 50 untrained individuals, both male and female, aged between 18 and 50. The research protocol received approval from the Research Ethics Review Committee for Research Involving Human Research Participants, Group 1, Chulalongkorn University (COA No. 234/65), and all participants provided written informed consent before the evaluation. Utilizing a 9-point hedonic scale, ranging from 1 (disliked extremely) to 9 (liked extremely), the sensory analysis evaluated BGB juice and its fermentation at various concentrations. Samples were assigned random 3-digit numbers and presented to panelists in a sensory evaluation laboratory. The panelists were guided on the use of the hedonic scale, instructed to cleanse their palate with water and salted crackers between samples, and assessed for color, appearance, odor, taste, sweetness, sourness, off-flavor, and overall acceptability. The sensory test occurred in individual booths with ample ventilation, under white light, and at room temperature.

### 2.10. Statistical Analyses

The results were expressed as mean values ± SEM, *n* = 3. Data were analyzed using the SPSS 21 statistical program (SPSS, Inc., Chicago, IL, USA). To identify any significant differences among treatments, a one-way analysis of variance (ANOVA) was performed at a 95% confidence level. Duncan’s multiple range test was employed to discriminate among the means of the various factors. The paired *t*-test was used to compare the differences in parameters before and after fermentation. Visualizations were created using Sigma Plot (version 12.0) and GraphPad Prism (version 9.5.1). The principal component analysis and volcano plot were analyzed using R (version 4.2.3).

## 3. Results and Discussion

### 3.1. Alterations in Physicochemical Properties Following Fermentation of BGB

The results indicate a substantial decrease in the pH value of the fermented juice, as detailed in Table 1. Prior to fermentation, the initial pH of the BGB juice ranged from 5.24 to 5.37. After 24 h fermentation at all treatment concentrations, there was a noticeable decrease in pH, falling within the range of 3.87 to 3.93. Before the fermentation process, an inverse relationship was observed between the concentration of BGB and the pH value of the mixture. As the concentration of BGB increased, the pH exhibited a decreasing trend. This can be attributed to the inherent acidity of the BGB, primarily due to its content of organic acids [19]. The higher concentration of these acidic compounds contributed to a lower pH, rendering the mixture more acidic. Furthermore, the concentration of BGB had a directly proportional effect on the TSS, reducing sugars, and total carbohydrate content. With an increase in BGB concentration, a significant elevation in these parameters was observed. This can be ascribed to the natural sugars present in BGB, such as glucose and fructose [19], becoming more concentrated as the mixture thickened. The higher concentration of these saccharides contributed to an increase in TSS, reducing sugars, and total carbohydrate levels.

Concurrently, the concentration of lactic acid after 24 h fermentation exhibited a significant increase for all BGB concentrations, with values ranging from 2.66 ± 0.03 to 8.11 ± 0.67 mg/mL. This decline in pH and increase in lactic acid concentration can be attributed to the heightened production of lactic acid, a metabolic by-product of LGG via the homofermentative pathway [30]. Lactic acid is generated through the metabolic utilization of saccharides present in the BGB. Consequently, the fermentation process with LGG led to a noteworthy reduction in total soluble solids (TSS), as well as total and reducing sugar content across all BGB concentrations. These findings align with a previous study where the fermentation of goji berry juice (*Lycium barbarum* L.) occurred in the presence of various lactic acid bacteria mixtures. The results suggest that diverse bacterial strains directly metabolized reducing sugars, resulting in the production of lactic acid and other chemical compounds through a sequence of biochemical reactions [31]. However, the concentration of BGB did not exhibit a significant impact on the physicochemical properties of the mixture following the fermentation process, suggesting that the changes observed were predominantly influenced by the fermentation conditions rather than the initial BGB concentration.

The analysis of color parameters revealed a significant increase in L*, a*, and b* values across all concentrations of BGB before and after 24 h of fermentation (Table 1). The elevation in the L* value may be attributed to the breakdown of juice components, particularly anthocyanin pigments, during fermentation, resulting in a lighter color. Similar observations were reported in a previous study where pomelo fermentation by lactobacilli led to an increase in the lightness value, potentially enhancing consumer perception and acceptance of the product [32]. The increase in color intensity within the red hue (a*) can be linked to the acidic conditions produced during fermentation, conducive to the alteration in the formation of anthocyanin pigments. Theoretically, the chemical structure of anthocyanin is pH-dependent. Under acidic conditions, particularly at pH = 1, anthocyanin exists in the form of a flavylium cation, contributing to the production of red and purple colors. This elucidates the significant increase in a* values observed after fermentation, concurrently with the reduction in pH value [33]. As the pH increases within the range of 2–4, the quinoidal blue species becomes prevalent. Additionally, at a pH between 5 and 6, colorless carbinol pseudobase and a chalcone appear. Given that anthocyanin pigments exhibit a reddish color in acidic conditions.

### 3.2. Alterations in Viable Lactobacilli Following Fermentation of BGB

This study presents the changes in the viable count of lactobacilli before and after 24 h of fermentation, as detailed in Table 1. Before fermentation, the initial viable cell counts ranged from 7.43 to 7.46 log_10_ CFU/mL, with similar counts observed in all concentrations of BGB. Following 24 h fermentation, a significant increase in viable counts was noted, reaching 8.52, 8.75, and 8.74 log_10_ CFU/mL in BGB concentrations of 2.5%, 5%, and 10%, respectively. No significant variation was observed among the different concentrations.

Several factors influence the viability of probiotics including pH, titratable acidity, oxygen level, water activity, salt, and sugar content. Therefore, the presence of sugar in BGB may exert a substantial impact on the growth and viability of probiotic bacteria. As evidenced by the previous study, a relationship was observed between the survival of LGG in the presence of glucose and its ability to utilize sugars. Thus, LGG demonstrated an ability to utilize carbohydrate components, thereby contributing to its overall survivability [34].

Consequently, the findings suggest that BGB serves as a viable probiotic carrier for *L. rhamnosus* GG bacteria, exhibiting substantial proliferation within the juice matrix. The achieved viable cell counts surpassed the recommended threshold for conferring health benefits, falling within the range of log 6–log 7 of probiotic bacteria per mL or gram of food [35].

### 3.3. Alterations in Phytochemical Composition Following Fermentation of BGB

The impact of LGG fermentation on phytochemical compounds and antioxidant activities in BGB is outlined in Table 2. TPC, TAC, and TFC levels exhibited a concentration-dependent trend, peaking at a 10% concentration of BGB. While TPC levels remained constant after fermentation across all treatments, TFC levels showed a significant increase, and TAC experienced a significant decrease after fermentation. The consistent TPC trend aligns with findings from a study on camu-camu fruit, where TPC levels remained stable following a 72 h fermentation period [36]. However, a decline at 24 h was noted, potentially due to initial phenolic utilization and rearrangement into polymeric forms, followed by the release of soluble free phenolic compounds at 48 h.

The decline in anthocyanin observed may be attributed to the enzymatic fermentation process, particularly involving β-glucosidase. This enzymatic activity hydrolyses the β-1,4-glycosidic bond and leads to a decrease in detectable anthocyanins, facilitating their conversion into the main phenolic acid product [37]. This aligns with studies on red cabbage sprouts and blueberry/blackberry juices [38], where probiotic fermentation resulted in reduced anthocyanin content and an upgraded trend in certain phenolic acids such as syringic acid, ferulic acid, and gallic acid [39]. An increase in TFC across all treatments suggests flavonoid glycosides may undergo degradation during fermentation, or the production of flavonoids may arise from the degradation of complex polyphenols [40]. This is consistent with a study on jujube juice fermented with LGG and *L. plantarum-1*, where total flavonoid content significantly increased [41]. Further investigations into the kinetic changes in *L. rhamnosus* GG activity within BGB are warranted.

### 3.4. Alterations in Antioxidant Activities Following Fermentation of BGB

The antioxidant activities of BGB before and after fermentation are presented in Table 2, indicating a concentration-dependent increase, with the highest observed at 10% BGB. This variation is influenced by factors such as environmental conditions, fruit varieties, and extraction methods [19]. Flavonoids, polysaccharides, carotenoids, and AA-2βG contribute to BGB’s antioxidant activities, functioning through mechanisms like radical scavenging, metal chelation, and interactions with other antioxidants [42,43]. Despite these variations, no significant differences were observed in antioxidant activity parameters after fermentation. This consistent pattern aligns with the stability trend of TPC. Similar trends in TPC and the antioxidant activity during the fermentation of fruit matrices with potential probiotics have been reported. For instance, the fermentation of camu-camu fruit by lactic acid bacteria showed no changes in total soluble phenolic content and antioxidant activity [36]. In contrast, the fermentation of goji berry by multiple strains of probiotics resulted in a significant increase in TPC and antioxidant activity. Various antioxidant indexes correlated with the concentrations of both free and bound forms of phenolic compounds [31]. Therefore, the sustained high antioxidant capacity, potentially associated with health benefits, was maintained after fermentation with LGG.

### 3.5. Alterations in Biological Properties Following Fermentation of BGB

**Figure 1:**
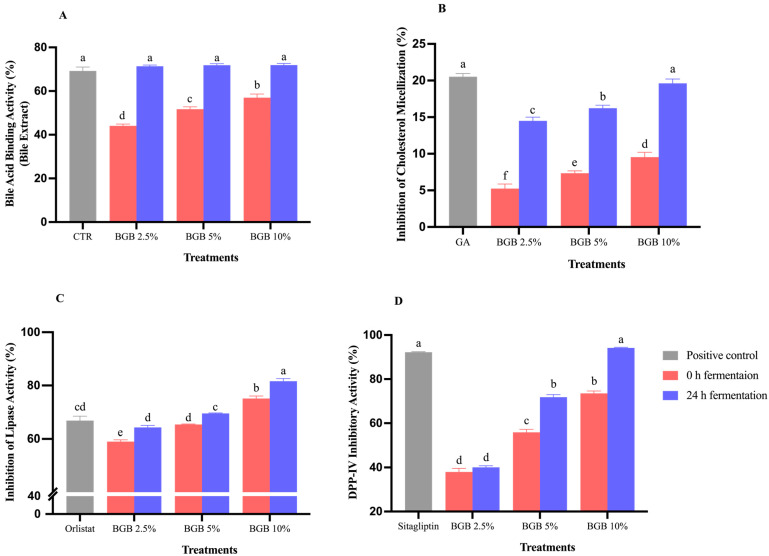
Potential biological activities of BGB beverage at 0 and 24 h of fermentation: The percentage of bile acid binding (**A**), the inhibition of cholesterol micellization (**B**), the inhibition of lipase activity (**C**), and DPP-IV inhibitory activity (**D**). The results were expressed as mean ± S.E.M. (*n* = 3). Significant differences are presented with different superscripted letters (*p* < 0.05). CTR: Cholestyramine; GA: Gallic acid.

The biological activity of fermented BGB, including bile acid binding, inhibition of cholesterol micellization, pancreatic lipase activity, and DPP-IV, is depicted in Figure 1. All BGB concentrations showed the ability to bind primary bile acid, with a significant increase of 38.24%, 28.03%, and 20.81% in BGB 2.5%, 5%, and 10% after fermentation (Figure 1A). The binding values of fermented samples were comparable to cholestyramine at a concentration of 3 mg/mL. This suggests that fermented BGB may disrupt the endogenous bile acid pool, potentially stimulating bile acid synthesis from cholesterol, and contributing to reduced blood cholesterol levels [44]. The phytochemical components of BGB, especially the increasing of flavonoids, might interact with bile acids through ionic, hydrogen, and hydrophobic interactions, forming insoluble polyphenol–bile acid complexes and increasing fecal bile excretion, disrupting micelle formation [44]. Additionally, a previous study observed a high bile acid adsorption capacity in flavonoid-rich lupin cotyledons (*Lupinus angustifolius* L.) and suggested the formation of hydrophobic interactions between polyphenols and bile acid [45].

In cholesterol absorption, micelles are formed in the intestine by bile salts, cholesterol, and phospholipids. Our study reveals a significant increase in the inhibition of cholesterol micellization formation after fermentation across all concentrations (Figure 1B). Similar findings were observed in the fermentation of gac fruit beverage with lactobacilli, demonstrating an increased capacity to disrupt cholesterol micellization formation [22]. In general, probiotics employ enzymes like hydroxysteroid dehydrogenase and conjugated bile acid hydrolase to break down bile acids and hydrolyze bile salts, disrupting the enterohepatic circulation of bile acids. Probiotic bacteria also reduce cholesterol absorption by binding it and incorporating it into the cell membrane, playing a preventive role in micelle production [46].

After fermentation, BGB significantly enhanced the concentration-dependent inhibition of pancreatic lipase across all treatments after fermentation (Figure 1C). This inhibitory effect may be attributed to polyphenols in BGB, forming complexes with pancreatic lipase and impairing its enzymatic activity [47]. Particularly, flavonoids have the potential to disrupt the structural integrity of lipase enzymes and diminish the substrate (olein) affinity for the enzyme. Consequently, the activity of the lipase was reduced [48]. Fermented BGB, through binding bile acid and inhibiting cholesterol micellization and pancreatic lipase activity, contributes to lipid-lowering effects. Fermented BGB exhibited concentration-dependent inhibition of DPP-IV, with notable increases observed at 5% and 10% BGB concentrations (Figure 1D). The inhibitory capability of fermented 10% BGB was comparable to sitagliptin (100 μM), an antidiabetic drug. This may result from hydrogen bonds formed between amino acid residues in DPP-IV and polyphenols present in fermented BGB [49]. Additionally, *Lactobacillus* spp. strains, especially *L. rhamnosus*, are reported to have DPP-IV inhibitory activity [50]. The fermentation of BGB contributes to enhanced DPP-IV inhibitory activity, a phenomenon modulated by the presence of bioactive components and the viability of probiotics. Theoretically, DPP-IV is an enzyme involved in the degradation of endogenous GLP-1. The inhibition effect on this enzyme may lead to an increase in active GLP-1, while concurrently reducing GLP-1 clearance. This results in a lowering of both fasting and postprandial glucose concentration, thereby offering potential benefits for individuals experiencing hyperglycemic conditions [51].

### 3.6. Effect of BGB on Cell Viability and Cholesterol Uptake in Caco-2 Cells

**Figure 2:**
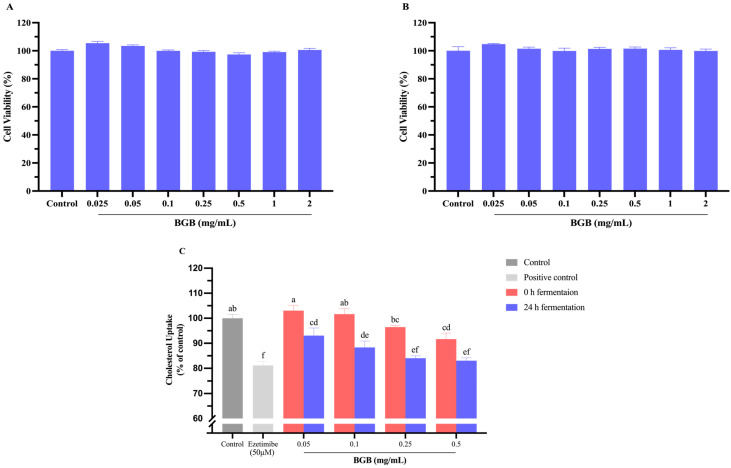
Effect of BGB on Caco-2 cells line: Cell viability after the treatment of cell with BGB at 0 h fermentation (**A**) and the treatment of cell with BGB at 24 h fermentation (**B**), and the impact of BGB on cholesterol uptake in Caco-2 cells (**C**). The results are presented as mean ± SEM (*n* = 3). Significant differences are presented with different superscripted letters (*p* < 0.05).

The MTT assay evaluated the cytotoxicity of non-fermented and fermented BGB on Caco-2 cells. As shown in Figure 2A,B, both samples exhibited no significant cytotoxicity at concentrations ranging from 0.025 to 2 mg/mL. The non-cytotoxic range identified was considered appropriate for subsequent cholesterol uptake assays, ensuring that any observed effects were not influenced by compromised cell viability.

The impact of BGB on the uptake of cholesterol into Caco-2 cells is illustrated in Figure 2C. Fermented BGB exhibited a significant reduction in cholesterol uptake compared to the baseline samples at 0 h. This reduction was observed in a concentration-dependent manner, with the highest reduction recorded at 16.94% with 0.5 mg/mL of fermented BGB. Interestingly, this reduction was comparable to the effect observed with 50 μM of Ezetimibe, a known cholesterol absorption inhibitor, which resulted in a reduction of 18.86%. The process of cholesterol uptake in cells is mediated by the Niemann–Pick C1-Like 1 (NPC1L1) protein transporter situated on the apical membrane of enterocytes [52]. Based on our findings, the observed increase in flavonoid content after fermentation may contribute to the reduction in cholesterol uptake in intestinal cells. Previous studies have demonstrated that pre-incubation of Caco-2 cells with flavonoids can lead to a concentration-dependent reduction in cholesterol uptake, thereby diminishing cholesterol absorption by influencing the intestinal epithelial cells [53]. LGG itself exhibits a capacity to reduce cholesterol uptake [54]. Furthermore, the bioconversion process during fermentation with LGG can alter the phytochemical composition of the samples. Notably, certain polyphenolic compounds have been reported to reduce cholesterol uptake by inhibiting the NPC1L1. These polyphenols include luteolin, quercetin, catechin, epigallocatechin gallate, and chlorogenic acid [55]. Interestingly, our analysis revealed an increase in the levels of these polyphenols after 24 h fermentation (Table S1).

### 3.7. Non-Volatile Compound Profiling 

**Figure 3:**
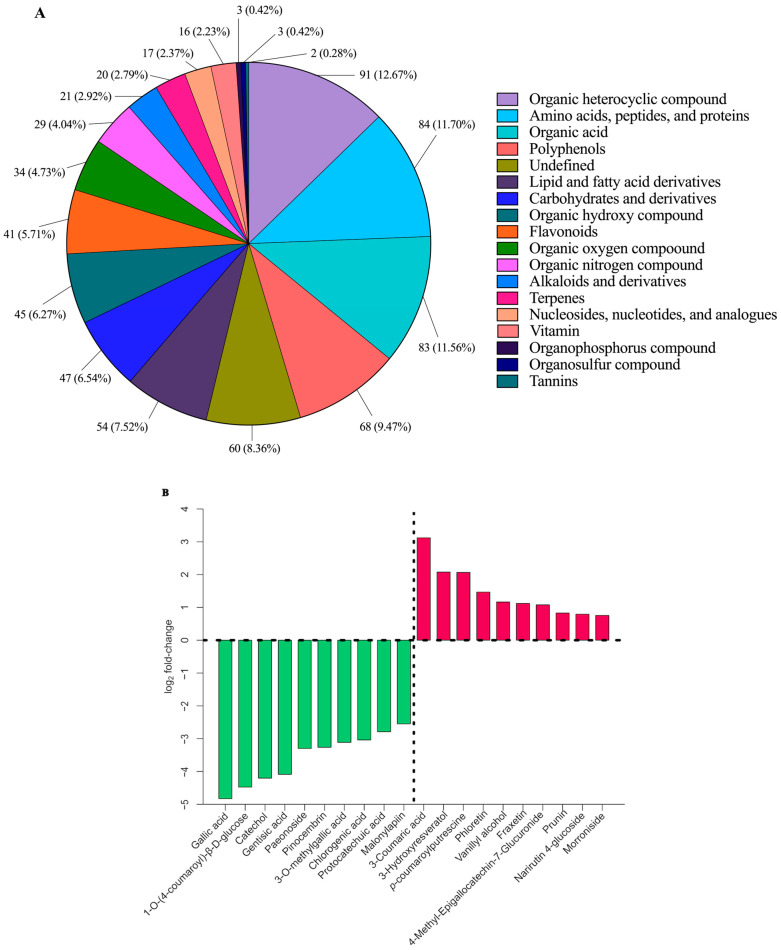
The quantitative distribution of identified metabolites across distinct chemical classes. Different color blocks represent different chemical classifications, the percentage denotes the proportion of metabolites within that classification relative to the overall metabolites. Unassigned metabolites were categorized as undefined (**A**) and Top 10 phytochemical compounds with the largest fold change. Green and red bars represent upregulated and downregulated metabolites after fermentation, respectively (**B**).

A total of 718 compounds were detected in the samples by Reverse-Phase High-Performance Liquid Chromatography–High-Resolution Fourier Transform Mass Spectrometry (HPLC-HRFTMS). Fold changes (expressed as Log_2_ fold changes) and ratios between the samples before and after fermentation were calculated to allow comparisons among samples. Adjusted *p*-values were also calculated to allow for statistical comparisons between the two samples. Based on our analysis using LCMS/MS, we detected a total of 339 and 379 metabolites in negative- and positive-ion modes, respectively. These metabolites belong to various classes of chemical compounds, as classified based on chemical taxonomy. The phytochemical profile of BGB comprises an array of bioactive compounds (Table S1), including flavonoids such as catechin, naringenin, and prunin, as well as anthocyanins, predominantly cyanidin. Additionally, BGB contains various phenolic acids, including gallic acid, gentisic acid, sinapinic acid, and caffeic acid. Figure 3A illustrates the quantitative distribution of the metabolites, with the top three chemical classes being organic heterocyclic compounds, amino acids, peptides, proteins, and organic acids. However, our primary focus was on investigating the correlation of phenolic compounds throughout the fermentation process.

Metabolites exhibiting significant differences (adjusted *p* < 0.05) are detailed in Table S1. The negative log_2_ fold change indicates an upregulation of compounds after 24 h of fermentation. Notably, a majority of the phenolics of interest (24 out of 44 compounds) demonstrate a significant increase after fermentation, including gallic acid, gentisic acid, chlorogenic acid, caffeic acid, sinapinic acid, protocatechuic acid, and hesperitin (Table S1). As depicted in Figure 3B, *p*-coumaric acid experienced the most pronounced decrease after BGB fermentation. This alteration is attributed to the activity of hydroxycinnamic reductase from *Lactobacillus* spp. These findings are consistent with previous studies on *L. plantarum* fermentation, which observed the conversion of phenolic acids (such as caffeic acid, *p*-coumaric acid, and ferulic acid) into caffeic acid, epicatechin, catechin, and rosmarinic acid [56]. 

**Figure 4:**
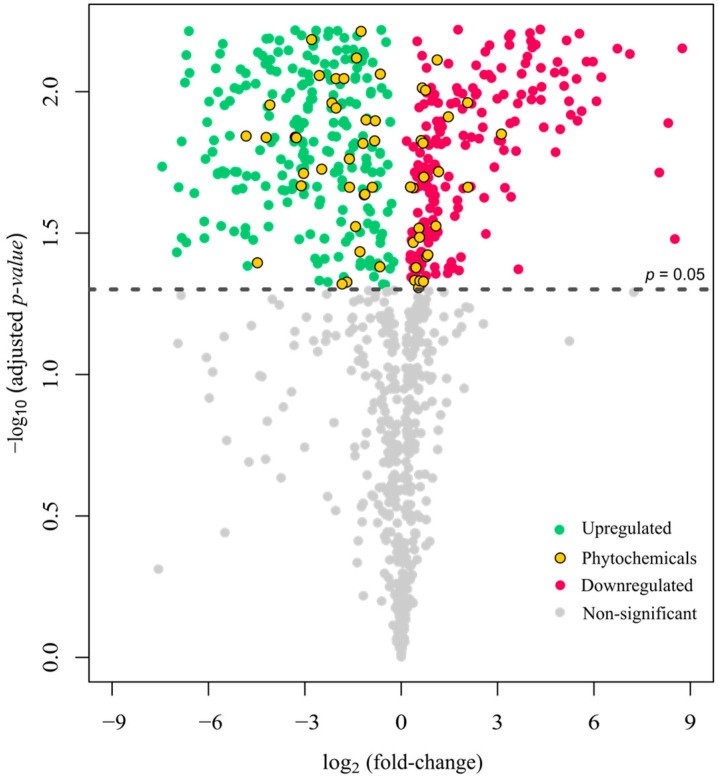
Volcano plot showing log_2_ fold change and adjusted *p*-values of metabolite compounds quantified using LC-HRFTMS. Negative log_2_ fold change indicates the increment of compounds. Green circles represent upregulated metabolites and red circles represent downregulated metabolites after fermentation. Yellow circles represent interested phenolic compounds.

Figure 4 highlights the interesting phenolic compounds with yellow circles. The increase in the fold change of phenolic acids and flavonoids can be attributed to the biotransformation properties exhibited by probiotics. These properties enable the utilization of phenolic compounds, which are subsequently converted into compounds often displaying greater bioactivity than their parent compounds [57]. While phenolic acids showed increasing trends after 24 h fermentation, other chemical compounds in BGB may be responsible for their antioxidant activities. Specifically, certain amino acids (tyrosine, tryptophan, methionine, lysine, cysteine, and histidine) and dipeptides containing these amino acid moieties could contribute significantly to the observed antioxidant effects. Antioxidant-active peptides typically consist of 5–16 amino acid residues and are known to inhibit lipid peroxidation, scavenge free radicals, and chelate transition metal ions [58]. Our findings indicated a reduction in certain antioxidant-active amino acids and peptides, such as tyrosine, tryptophan, and valine-tryptophan (Table S1). This alteration may be attributed to the utilization of the LGG where amino acids are potentially utilized as an energy source [59]. Consequently, the consistent antioxidant activities observed after bacteria fermentation may be attributed to the decrease in specific amino acids and the concurrent increase in phenolic contents, including phenolic acids and flavonoids. It is plausible that the balanced increase and decrease in these compounds contribute to the maintenance of antioxidant activity levels despite individual fluctuations.

Additionally, a decrease in anthocyanin was observed after BGB fermentation. This could be attributed to the degradation of anthocyanin glucoside into its aglycone forms and free phenolic acids. Similar findings were reported in a previous study, where fermentation of jussara pulp by lactobacilli led to the conversion of cyanidin-3-glucoside and cyanidin-3-rutinoside into the main phenolic acid product, protocatechuic acid [60]. Furthermore, phenolic metabolites might influence the growth and metabolism of probiotics. A previous study showed that the flavanol catechin stimulated the growth of *L. plantarum* by facilitating expedited sugar consumption, increasing sugar utilization, and triggering malic acid decarboxylation [61]. Therefore, alterations in phenolic components could potentially stimulate the growth of probiotics in BGB.

### 3.8. Sensory Evaluation

The sensory evaluation of BGB samples across various attributes is depicted in Table S2. BGB presents a unique blend of sweet, tangy, and pungent flavors, making it highly attractive to consumers. However, there is an inverse correlation between acceptability scores and BGB concentration. Higher concentrations of BGB resulted in a significant reduction in almost all evaluated attributes, including appearance, color, taste, sweetness, sourness, off-flavor, and overall acceptability. This trend may be attributed to the concentration-dependent nature of phenolic compounds (TPC, TFC, and TAC) in BGB samples. Polyphenols also contribute to sensory characteristics such as color, flavor, odor, astringency, and bitterness [62]. Consequently, the increased presence of phenolic components in BGB may be associated with perceptions of bitterness and astringency, leading to reduced consumer acceptance.

Conversely, sensory acceptability significantly improved after fermentation. The 10% BGB initially obtained the lowest acceptability score before fermentation. However, after fermentation, its attribute scores improved and became comparable to those of the 2.5% and 5% BGB across all parameters. Due to lactic acid production by lactobacilli, the sourness score exhibited a significant increase. In essence, the fermentation of BGB has the potential to enhance sensory acceptability scores across various attributes, including appearance, color, odor, taste, sweetness, sourness, off-flavor, and overall acceptability.

### 3.9. Principal Component Analysis (PCA)

**Figure 5:**
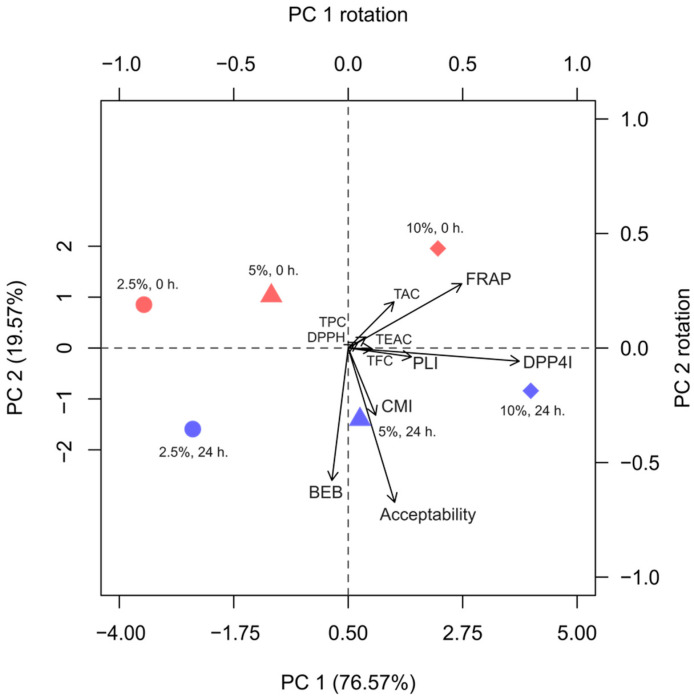
Principal component analysis (PCA) and biplot of BGB samples at 0 and 24 h of fermentation. Principal axes were calculated from 3 groups of parameters: phytochemical, biological, and sensory acceptability. TPC: Total phenolic content; TFC: Total flavonoid content; TAC: Total anthocyanin content; DPPH: DPPH radical scavenging activity; FRAP: Ferric-reducing antioxidant power; TEAC: Trolox equivalent antioxidant capacity; CMI: Cholesterol micellization inhibition; PLI: Pancreatic lipase inhibition; BEB: Bile extract binding; DPP4I: Dipeptidyl peptidase-IV inhibition.

The principal component analysis (PCA) depicted in Figure 5 reveals that two principal components (PCs) accounted for over 95% of the variance. PC1 explained 76.6% and PC2 explained 19.6% of the total sample variance. Along PC1, variance was primarily associated with sample concentration, while along PC2, variance reflected changes in parameters between 0 and 24 h fermentation. Variable loadings and the biplot indicated that DPP-IV inhibition and FRAP were the key parameters contributing to PC1. The concentration of BGB positively correlated with DPP-IV inhibition and FRAP. Conversely, bile extract binding ability and sensory acceptability were the main contributors to PC2, albeit in a partially opposite direction to FRAP. Fermented samples exhibited higher bile acid binding and sensory acceptability, as depicted in Figure 1A and Table S2. This analysis reinforces the observed trends in parameter concentrations and the impact of fermentation.

## 4. Conclusions

BGB is an effective carrier for LGG, enhancing lactic acid production and probiotic viability over 24 h of fermentation. Fermentation reduced pH, total soluble solids (TSS), total carbohydrates, and reducing sugars, turning BGB purple due to increased acidity. Biochemically, TPC remained stable, maintaining consistent antioxidant activity. However, TFC increased, while TAC decreased post-fermentation. The fermentation process alters BGB’s chemical composition, resulting in heightened levels of phenolic acids like gallic acid and chlorogenic acid, as well as flavonoids, amino acids, and dipeptides. Notably, fermented BGB exhibits enhanced functional properties, including improved bile extract binding, inhibition of cholesterol micellization and pancreatic lipase, and decreased cholesterol uptake in intestinal cells without any cytotoxic effects. These findings suggest potential benefits for managing hyperlipidemia by targeting lipid digestion and absorption. Additionally, fermented BGB shows promise in inhibiting dipeptidyl peptidase-IV (DPP-IV), indicating its potential role in managing hyperglycemia. Additionally, fermented BGB showed an improvement in sensory quality. Overall, these results highlight the potential of LGG fermentation to enhance the functional properties of BGB, paving the way for the development of innovative functional foods aimed at addressing hyperlipidemia and hyperglycemia.

## Figures and Tables

**Table 1:** Physiochemical parameters of different concentrations of BGB juice at 0 and 24 h fermentation.

Samples	pH	TSS (^o^Brix)	Total Sugar (mg/mL)	Reducing Sugar (mg/mL)	Lactic Acid (mg/mL)	L*	a*	b*	Bacterial Enumeration (LogCFU/mL)
BGB 2.5%, 0 h	5.37 ± 0.01 ^Aa^	7.88 ± 0.02 ^Ca^	65.84 ± 1.21 ^Ca^	21.46 ± 0.17 ^Ca^	N.D.	0.77 ± 0.08 ^Ab^	0.45 ± 0.06 ^Ab^	0.43 ± 0.05 ^Bb^	7.43 ± 0.01 ^Bb^
BGB 2.5%, 24 h	3.88 ± 0.01 ^Bb^	7.56 ± 0.01 ^Cb^	57.31 ± 1.41 ^Cb^	19.60 ± 0.26 ^Cb^	2.66 ± 0.03 ^C^	1.95 ± 0.10 ^Aa^	3.30 ± 0.06 ^Aa^	1.07 ± 0.06 ^Aa^	8.52 ± 0.07 ^Aa^
BGB 5%, 0 h	5.32 ± 0.01 ^Ba^	10.22 ± 0.03 ^Ba^	73.64 ± 0.92 ^Ba^	46.48 ± 0.51 ^Ba^	N.D.	0.33 ± 0.03 ^Bb^	0.16 ± 0.03 ^Bb^	0.24 ± 0.05 ^Ab^	7.46 ± 0.01 ^Bb^
BGB 5%, 24 h	3.87 ± 0.00 ^Bb^	9.86 ± 0.03 ^Bb^	65.39 ± 1.43 ^Bb^	40.08 ± 0.05 ^Bb^	5.17 ± 0.35 ^B^	0.78 ± 0.01 ^Ba^	2.10 ± 0.03 ^Ba^	0.55 ± 0.03 ^Ba^	8.75 ± 0.02 ^Aa^
BGB 10%, 0 h	5.24 ± 0.01 ^Ca^	14.36 ± 0.05 ^Aa^	92.36 ± 1.31 ^Aa^	95.37 ± 1.38 ^Aa^	N.D.	0.21 ± 0.02 ^Bb^	0.07 ± 0.09 ^Bb^	0.25 ± 0.05 ^Ab^	7.45 ± 0.00 ^Bb^
BGB 10%, 24 h	3.93 ± 0.01 ^Ab^	13.90 ± 0.06 ^Ab^	80.27 ± 1.12 ^Ab^	82.88 ± 0.46 ^Ab^	8.11 ± 0.67 ^A^	0.54 ± 0.01 ^Ca^	1.35 ± 0.11 ^Ca^	0.27 ± 0.01 ^Ca^	8.74 ± 0.08 ^Aa^

The results are expressed as mean ± S.E.M. (*n* = 3). Means with different uppercase letters at the same time point (A–C: treatment effects) and lowercase letters at the same treatment (a,b: time effects) are significantly different (*p* < 0.05). BGB: Black Goji Berry extract; TSS: Total Soluble Solids; N.D.: Not detected.

**Table 2:** Biochemical and Antioxidant parameters of different concentrations of BGB beverage at 0 and 24 h fermentation.

Samples	TPC (g GAE/100 mL)	TAC (mg C3GE/100 mL)	TFC (mg QE/100 mL)	FRAP (mmol FeSO_4_/100 mL)	DPPH (mg AAE/100 mL)	TEAC (mg TE/100 mL)
BGB 2.5%, 0 h	0.13 ± 0.00 ^Ca^	6.84 ± 0.12 ^Ca^	2.21 ± 0.16 ^Cb^	1.18 ± 0.02 ^Ca^	67.55 ± 1.92 ^Ca^	637.70 ± 27.94 ^Ca^
BGB 2.5%, 24 h	0.13 ± 0.00 ^Ca^	6.17 ± 0.07 ^Cb^	4.43 ± 0.11 ^Ca^	1.22 ± 0.02 ^Ca^	68.37 ± 1.92 ^Ca^	637.22 ± 18.94 ^Ca^
BGB 5%, 0 h	0.25 ± 0.00 ^Ba^	12.77 ± 0.10 ^Ba^	5.76 ± 0.25 ^Bb^	2.36 ± 0.04 ^Ba^	119.88 ± 0.88 ^Ba^	810.99 ± 30.59 ^Ba^
BGB 5%, 24 h	0.25 ± 0.00 ^Ba^	11.40 ± 0.11 ^Bb^	7.75 ± 0.21 ^Ba^	2.38 ± 0.02 ^Ba^	121.42 ± 2.43 ^Ba^	809.17 ± 18.92 ^Ba^
BGB 10%, 0 h	0.41 ± 0.00 ^Aa^	22.79 ± 0.28 ^Aa^	9.42 ± 0.05 ^Ab^	4.47 ± 0.01 ^Aa^	230.77 ± 5.30 ^Ab^	1158.33 ± 22.69 ^Aa^
BGB 10%, 24 h	0.42 ± 0.00 ^Aa^	18.92 ± 0.09 ^Ab^	10.36 ± 0.18 ^Aa^	4.59 ± 0.05 ^Aa^	240.19 ± 3.31 ^Aa^	1160.86 ± 15.55 ^Aa^

The results are expressed as mean ± S.E.M. (*n* = 3). Means with different uppercase letters at the same time point (A–C: treatment effects) and lowercase letters at the same treatment (a,b: time effects) are significantly different (*p* < 0.05). TPC: Total phenolic content; TAC: Total Anthocyanin content; TFC: Total Flavonoid content; FRAP: Ferric-Reducing Antioxidant Power; DPPH: DPPH radical scavenging activity; TEAC: Trolox Equivalent Antioxidant Activity; GAE: Gallic acid equivalent; C3GE: Cyanidin-3-glucoside equivalent; QE: Quercetin equivalent; AAE: Ascorbic Acid Equivalent; TE: Trolox Equivalent.

## Data Availability

Data are contained within the article and Supplementary Materials.

## Supplementary Materials

The following supporting information can be downloaded at: https://www.mdpi.com/article/10.3390/antiox13060740/s1, Table S1: Non-volatile compounds identified in BGB beverage at 0 and 24 h of fermentation; Table S2: Sensory acceptability of BGB beverage at 0 and 24 h of fermentation.
