# Supplementary material for: Black Goji Berry (Lycium ruthenicum) Juice Fermented with Lactobacillus rhamnosus GG Enhances Inhibitory Activity against Dipeptidyl Peptidase-IV and Key Steps of Lipid Digestion and Absorption
Source: Antioxidants (Basel). 2024 Jun 19;13(6):740. doi: 10.3390/antiox13060740 (PMC11200685; doi:10.3390/antiox13060740)
Supplement: Supplementary file 1 [file antioxidants-13-00740-s001.zip › antioxidants-3042552-supplementary.pdf]

# **Black Goji Berry (*Lycium ruthenicum*) Juice Fermented with *Lactobacillus rhamnosus* GG Enhances Inhibitory Activity Against Dipeptidyl Peptidase-IV and Key Steps of Lipid Digestion and Absorption.**

Kritmongkhon Kamonsuwan[1], Vernabelle Balmori[2], Marisa Marnpae[3], Charoonsri Chusak[1], Thavaree Thilavech[4], Suvimol Charoensiddhi[5], Scott Smid[6], Sirichai Adisakwattana[*,1]

[1] Center of Excellence in Phytochemical and Functional Food for Clinical Nutrition, Department of Nutrition and Dietetics, Faculty of Allied Health Science, Chulalongkorn University, Bangkok, 10330, Thailand.

[2] Department of Food Science and Technology, Southern Leyte State University, Sogod 6606, Southern Leyte, Philippines.

[3] The Halal Science Center, Chulalongkorn University, Bangkok, 10330, Thailand.

[4] Department of Food Chemistry, Faculty of Pharmacy, Mahidol University, Bangkok, 10400, Thailand.

[5] Department of Food Science and Technology, Faculty of Agro-Industry, Kasetsart University, Bangkok, 10900, Thailand.

[6] Discipline of Pharmacology, School of Biomedicine, Faculty of Health and Medical Sciences, The University of Adelaide, South Australia, 5000, Australia.

* Corresponding authors

Professor Sirichai Adisakwattana

Email: Sirichai.a@chula.ac.th, Tel: (+66) 2-218-1099 ext. 111

**Table S1.** Non-volatile compounds identified in BGB beverage at 0 and 24 h of fermentation.

| | Metabolites | Formula | RT (min) | Peak Areas (x10$^{6}$) | | $Log_2$ Fold change | Adj. *p*-value | Changes |
|---|---|---|---|---|---|---|---|---|
| | | | | 0 h. fermentation | 24 h. fermentation | | | |
| | **Phenolic acids** | | | | | | | |
| 1. | 3-Coumaric acid | $C_9 H_{18} N_2 O_3$ | 1.62 | 5.38 ± 0.30 | 0.62 ± 0.02 | 3.12 | 0.01 | Down |
| 2. | Gallic acid | $C_7 H_6 O_5$ | 1.94 | 0.71 ± 0.01 | 20.23 ± 1.00 | -4.83 | 0.01 | Up |
| 3. | Gentisic acid | $C_7 H_6 O_4$ | 3.30 | 3.45 ± 0.10 | 58.71 ± 2.12 | -4.09 | 0.01 | Up |
| 4. | Chlorogenic acid | $C_{16} H_{18} O_9$ | 3.44 | 0.43 ± 0.01 | 3.50 ± 0.26 | -3.04 | 0.02 | Up |
| 5. | Protocatechuic acid | $C_7 H_6 O_4$ | 3.46 | 0.58 ± 0.05 | 3.97 ± 0.04 | -2.79 | 0.01 | Up |
| 6. | Salicylic acid | $C_7 H_6 O_3$ | 3.57 | 35.65 ± 1.35 | 27.66 ± 0.73 | 0.37 | 0.02 | Down |
| 7. | p-coumaroylputrescine | $C_{13} H_{18} N_2 O_2$ | 5.36 | 1.74 ± 0.05 | 0.42 ± 0.01 | 2.07 | 0.01 | Down |
| 8. | 3-O-methylgallic acid | $C_8 H_8 O_5$ | 6.24 | 23.03 ± 1.25 | 199.28 ± 16.06 | -3.11 | 0.02 | Up |
| 9. | Vanillic acid 4-β-D-glucoside | $C_{14} H_{18} O_9$ | 6.76 | 6.06 ± 0.12 | 4.68 ± 0.10 | 0.37 | 0.03 | Down |
| 10. | Dihydrocaffeic acid | $C_9 H_{10} O_4$ | 7.88 | 16.31 ± 0.36 | 66.69 ± 1.42 | -2.03 | 0.01 | Up |
| 11. | Caffeic acid 3-glucoside | $C1_5 H_{18} O_9$ | 10.56 | 10.33 ± 0.30 | 22.04 ± 0.76 | -1.09 | 0.01 | Up |
| 12. | 1-O-(4-coumaroyl)-beta-D-glucose | $C_{15} H_{18} O_8$ | 10.87 | 3.64 ± 0.42 | 81.00 ± 11.78 | -4.48 | 0.04 | Up |
| 13. | Caffeic acid | $C_9 H_8 O_4$ | 10.98 | 94.13 ± 43.65 | 303.99 ± 15.27 | -1.69 | 0.05 | Up |
| 14. | 1-Caffeoyl-beta-D-glucose | $C_{15} H_{18} O_9$ | 12.46 | 10.50 ± 0.15 | 42.88 ± 0.64 | -2.03 | 0.01 | Up |
| 15. | 1-O-feruloyl-beta-D-glucose | $C_{16} H_{20} O_9$ | 12.97 | 36.49 ± 0.68 | 26.53 ± 1.22 | 0.46 | 0.04 | Down |
| 16. | Isoferulic acid | $C_{10} H_{10} O_4$ | 18.22 | 1.25 ± 0.44 | 6.96 ± 0.05 | -2.47 | 0.02 | Up |
| 17. | Sinapoylglucose | $C_{17} H_{22} O_{10}$ | 19.88 | 8.03 ± 0.22 | 5.41 ± 0.37 | 0.57 | 0.03 | Down |
| 18. | Sinapinic acid | $C_{11} H_{12} O_5$ | 20.99 | 47.98 ± 1.47 | 125.73 ± 1.62 | -1.39 | 0.01 | Up |
| 19. | Isochlorogenic acid | $C_{25} H_{24} O_{12}$ | 26.85 | 22.05 ± 1.09 | 16.50 ± 0.57 | 0.42 | 0.05 | Down |

The results are expressed as mean ± S.E.M. (n = 3).

**Table S1.** Non-volatile compounds identified in BGB beverage at 0 and 24 h of fermentation (Cont'd)

| | Metabolites | Formula | RT (min) | Peak Areas (x10$^6$) | | Log$_2$ Fold change | Adj. $p$-value | Changes |
|---|---|---|---|---|---|---|---|---|
| | | | | 0 h. fermentation | 24 h. fermentation | | | |
| | **Flavonoids** | | | | | | | |
| 20. | Gallocatechin | $C_{15} H_{14} O_7$ | 6.40 | 2.04 ± 0.03 | 5.46 ± 0.42 | -1.42 | 0.03 | Up |
| 21. | kaempferol 3,7-bis-O-β-d-glucopyranoside | $C_{15} H_{20} O_8$ | 9.58 | 6.19 ± 0.83 | 60.96 ± 3.62 | -3.30 | 0.01 | Up |
| 22. | Catechin | $C_{15} H_{14} O_6$ | 9.74 | 13.45 ± 0.22 | 23.53 ± 0.64 | -0.81 | 0.01 | Up |
| 23. | Malonylapiin | $C_{29} H_{30} O_{17}$ | 11.95 | 0.70 ± 0.05 | 4.08 ± 0.15 | -2.55 | 0.01 | Up |
| 24. | 4'-Methyl-Epigallocatechin-7-Glucuronide | $C_{22} H_{24} O_{13}$ | 25.65 | 3.18 ± 0.14 | 1.50 ± 0.09 | 1.08 | 0.03 | Down |
| 25. | Narirutin-4-glucoside | $C_{33} H_{42} O_{19}$ | 26.03 | 12.21 ± 0.15 | 7.60 ± 0.17 | 0.68 | 0.02 | Down |
| 26. | Cyanidin | $C_{15} H_{10} O_6$ | 26.67 | 2.55 ± 0.03 | 2.08 ± 0.02 | 0.29 | 0.02 | Down |
| 27. | Phloridzin | $C_{21} H_{24} O_{10}$ | 26.97 | 3.17 ± 0.05 | 2.13 ± 0.12 | 0.58 | 0.05 | Down |
| 28. | liquiritigenin | $C_{15} H_{12} O_4$ | 27.09 | 2.41 ± 0.05 | 1.57 ± 0.02 | 0.62 | 0.01 | Down |
| 29. | Prunin | $C_{21} H_{22} O_{10}$ | 27.13 | 13.96 ± 0.40 | 7.87 ± 1.17 | 0.83 | 0.04 | Down |
| 30. | Phloretin | $C_{15} H_{14} O_5$ | 27.90 | 1.57 ± 0.01 | 0.57 ± 0.01 | 1.47 | 0.01 | Down |
| 31. | Naringenin | $C_{15} H_{12} O_5$ | 28.45 | 18.20 ± 0.66 | 28.83 ± 1.33 | -0.66 | 0.04 | Up |
| 32. | Hesperetin | $C_{16} H_{14} O_6$ | 28.80 | 0.63 ± 0.03 | 1.44 ± 0.05 | -1.19 | 0.02 | Up |
| 33. | Isorhamnetin | $C_{16} H_{12} O_7$ | 29.57 | 1.96 ± 0.06 | 3.64 ± 0.14 | -0.90 | 0.02 | Up |
| 34. | pinocembrin | $C_{15} H_{12} O_4$ | 30.24 | 0.89 ± 0.02 | 8.52 ± 0.39 | -3.26 | 0.01 | Up |
| | **Other Phenolics** | | | | | | | |
| 35. | Vanillyl alcohol | $C_8 H_{10} O_3$ | 3.19 | 2.01 ± 0.04 | 0.90 ± 0.07 | 1.16 | 0.02 | Down |
| 36. | Catechol | $C_6 H_6 O_2$ | 3.30 | 0.22 ± 0.03 | 4.08 ± 0.20 | -4.20 | 0.01 | Up |
| 37. | Arbutin | $C_{12} H_{16} O_7$ | 6.07 | 0.55 ± 0.04 | 1.67 ± 0.04 | -1.61 | 0.02 | Up |
| 38. | 7-Hydroxycoumarine | $C_9 H_6 O_3$ | 6.20 | 35.69 ± 1.95 | 127.95 ± 11.97 | -1.84 | 0.05 | Up |
| 39. | Tyrosol | $C_8 H_{10} O_2$ | 7.89 | 0.41 ± 0.01 | 1.82 ± 0.06 | -2.16 | 0.01 | Up |
| 40. | vanilloloside | $C_{14} H_{20} O_8$ | 9.96 | 75.26 ± 0.77 | 46.15 ± 1.66 | 0.71 | 0.02 | Down |
| 41. | Liqcoumarin | $C_{12} H_{10} O_4$ | 12.97 | 2.15 ± 0.09 | 1.33 ± 0.08 | 0.69 | 0.05 | Down |
| 42. | morroniside | $C_{17} H_{26} O_{11}$ | 21.77 | 9.31 ± 0.13 | 5.51 ± 0.16 | 0.76 | 0.01 | Down |
| 43. | Fraxetin | $C_{10} H_8 O_5$ | 25.65 | 3.47 ± 0.08 | 1.59 ± 0.02 | 1.12 | 0.01 | Down |
| 44. | 3-Hydroxyresveratol | $C_{14} H_{12} O_4$ | 27.60 | 1.55 ± 0.11 | 0.37 ± 0.04 | 2.08 | 0.02 | Down |

The results are expressed as mean ± S.E.M. (n = 3).

**Table S1.** Non-volatile compounds identified in BGB beverage at 0 and 24 h of fermentation (Cont'd)

| | Metabolites | Formula | RT (min) | Peak Areas (x10$^6$) | | Log$_2$ Fold change | Adj. *p*-value | Changes |
|---|---|---|---|---|---|---|---|---|
| | | | | 0 h. fermentation | 24 h. fermentation | | | |
| | **Amino and Dipeptides** | | | | | | | |
| 44. | L-tyrosine | $C_9 H_{11} N O_3$ | 1.27 | 21.97 ± 2.89 | 8.25 ± 1.45 | 1.41 | 0.13 | Down |
| 45. | L-tryptophan | $C_{11} H_{12} N_2 O_2$ | 5.31 | 1,010.51 ± 22.37 | 733.92 ± 22.89 | 0.46 | 0.05 | Down |
| 46. | Valine-Leucine | $C_{11} H_{22} N_2 O_3$ | 4.69 | 2.80 ± 0.09 | 0.18 ± 0.01 | 3.93 | 0.01 | Down |
| 47. | Valine-Phenylalanine | $C_{14} H_{20} N_2 O_3$ | 13.79 | 3.18 ± 0.03 | 0.09 ± 0.02 | 5.07 | 0.01 | Down |
| 48. | Leucine-Leucine | $C_{12} H_{24} N_2 O_3$ | 18.28 | 2.33 ± 0.03 | 0.08 ± 0.01 | 4.87 | 0.01 | Down |
| 49. | Isoleucine-Isoleucine | $C_{12} H_{24} N_2 O_3$ | 15.71 | 36.45 ± 1.21 | 0.26 ± 0.02 | 7.12 | 0.01 | Down |
| 50. | Valine-Tryptophan | $C_{16} H_{21} N_3 O_3$ | 18.61 | 8.63 ± 0.16 | 0.42 ± 0.03 | 4.37 | 0.01 | Down |
| 51. | Leucine-Phenylalanine | $C_{15} H_{22} N_2 O_3$ | 22.70 | 48.18 ± 2.06 | 3.59 ± 0.15 | 3.74 | 0.01 | Down |

The results are expressed as mean ± S.E.M. (n = 3).

**Table S2.** Sensory acceptability of BGB beverage at 0 and 24 h of fermentation

| Samples | Appearance | Color | Odor | Taste | Sweetness | Sourness | Off-flavor | Overall acceptability |
|---|---|---|---|---|---|---|---|---|
| BGB 2.5%, 0 h | $6.58 \pm 0.23^{ab}$ | $6.06 \pm 0.27^{bc}$ | $5.54 \pm 0.25^{a}$ | $5.34 \pm 0.30^{a}$ | $5.52 \pm 0.29^{a}$ | $4.88 \pm 0.30^{bc}$ | $5.06 \pm 0.26^{a}$ | $5.68 \pm 0.29^{ab}$ |
| BGB 2.5%, 24 h | $6.92 \pm 0.22^{a}$ | $6.74 \pm 0.22^{ab}$ | $5.02 \pm 0.26^{a}$ | $5.88 \pm 0.29^{a}$ | $5.86 \pm 0.28^{a}$ | $5.70 \pm 0.32^{ab}$ | $5.22 \pm 0.25^{a}$ | $6.20 \pm 0.26^{a}$ |
| BGB 5%, 0 h | $6.14 \pm 0.25^{bc}$ | $6.18 \pm 0.25^{bc}$ | $5.60 \pm 0.23^{a}$ | $4.36 \pm 0.30^{b}$ | $4.52 \pm 0.27^{b}$ | $4.48 \pm 0.30^{cd}$ | $4.22 \pm 0.24^{b}$ | $4.96 \pm 0.29^{b}$ |
| BGB 5%, 24 h | $6.98 \pm 0.20^{a}$ | $7.10 \pm 0.21^{a}$ | $5.00 \pm 0.25^{a}$ | $5.78 \pm 0.28^{a}$ | $5.48 \pm 0.25^{a}$ | $5.60 \pm 0.29^{ab}$ | $5.08 \pm 0.26^{a}$ | $6.14 \pm 0.25^{a}$ |
| BGB 10%, 0 h | $5.68 \pm 0.26^{c}$ | $5.50 \pm 0.29^{c}$ | $5.10 \pm 0.22^{a}$ | $3.50 \pm 0.27^{c}$ | $3.74 \pm 0.26^{c}$ | $3.82 \pm 0.26^{d}$ | $3.32 \pm 0.24^{c}$ | $3.78 \pm 0.27^{c}$ |
| BGB 10%, 24 h | $7.12 \pm 0.20^{a}$ | $7.20 \pm 0.20^{a}$ | $5.22 \pm 0.23^{a}$ | $5.48 \pm 0.26^{a}$ | $5.34 \pm 0.24^{a}$ | $5.80 \pm 0.23^{a}$ | $4.66 \pm 0.21^{ab}$ | $6.06 \pm 0.21^{a}$ |

Values are expressed as mean ± S.E.M. (n=50). Means with different lowercase letters at the same time point (a-d: treatment effects) are significantly different ($p < 0.05$).
